# Suspected Acute Pulmonary Coccidioidomycosis in Traveler Returning to Switzerland from Peru

**DOI:** 10.3201/eid3011.241034

**Published:** 2024-11

**Authors:** Andreas Neumayr, Volker Rickerts, Sina Ackermann, Felipe Castelblanco, Esther Kuenzli, Ana Durovic, Carlos Seas

**Affiliations:** James Cook University, Townsville, Queensland, Australia (A. Neumayr); Swiss Tropical and Public Health Institute, Basel, Switzerland (A. Neumayr, E. Kuenzli, A. Durocic); University of Basel, Basel (A. Neumayr, E. Kuenzli, A. Durocic); Robert Koch Institute, Berlin, Germany (V. Rickerts, S. Ackermann); Institute Art Gender Nature Basel Academy of Art and Design FHNW, Basel (F. Castelblanco); Universidad Peruana Cayetano Heredia, Lima, Peru (C. Seas); Hospital Cayetano Heredia, Lima (C. Seas).

**Keywords:** coccidioidomycosis, coccidioides, Valley Fever, fungi, fungal, mycosis, Peru, Switzerland

## Abstract

We report a suspected case of acute pulmonary coccidioidomycosis contracted in Peru, where the disease is not known to occur, in a patient from Switzerland. Although not confirmed by direct diagnostic testing, the clinical manifestations and serologic testing results of this case are highly suggestive of coccidioidomycosis.

In November 2022, a 37-year-old man from Switzerland was referred by his general practitioner for further evaluation of an unknown febrile illness with respiratory symptoms. The patient had returned 3 weeks earlier from Peru, where he spent several days touring the southern coastal region, Ica, and Nazca and spent multiple weeks in the southeastern Amazon Basin region of Peru conducting field research in September 2022. The patient experienced an acute febrile illness with headache, myalgia, night sweats, dry cough, and dyspnea beginning 10 days after his return to Switzerland. The patient’s general practitioner ruled out malaria and dengue fever and referred him to the Swiss Tropical and Public Health Institute (Basel, Switzerland) when symptoms did not improve over a 2-week period. 

The patient’s initial physical examination was unremarkable. Laboratory testing revealed an unremarkable complete blood count and creatinine level but found elevated C-reactive protein level of 46 mg/L (reference range <5 mg/L), aspartate aminotransferase level of 99 U/L (reference range <40 U/L), alanine aminotransferase level of 142 U/L (reference range <40 U/L), gamma-glutamyl transferase level of 69 U/L (reference range <68 U/L), and lactate dehydrogenase level of 305 U/L (reference range <225 U/L). Results of blood cultures, HIV screening, interferon-γ release assay, *Histoplama capsulatum* immunodiffusion antibody test, *Cryptococcus* antigen test, *Coxiella burnetii* serologic test, and urine *H. capsulatum* and *Legionella pneumophila* antigen tests were negative. Computed tomography (CT) scan revealed a consolidation (sized 4.4 × 2 cm) in the right lower lung lobe (Figure 1), enlarged lymph nodes in the infracarinal region (3.8 × 2.3 cm), and less-pronounced enlarged lymph nodes (<1 cm) in the right hilar, hepatic hilar, celiac trunk, and retroperitoneal regions. We repeated the serologic testing 2 weeks later because of the possibility of delayed seroconversion in histoplasmosis cases, but results remained negative. 

**Figure 1:**
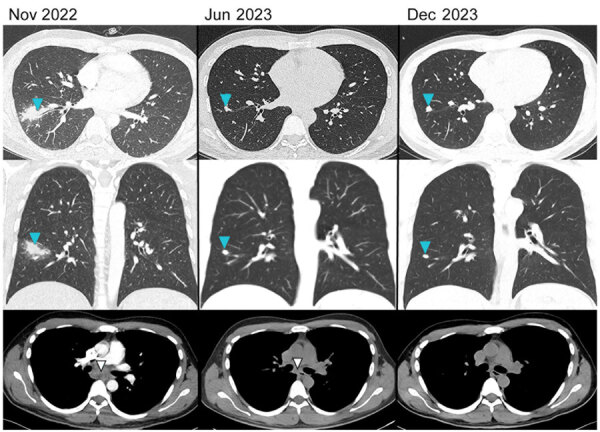
Computed tomography scan findings of a patient with a suspected case of coccidioidomycosis contracted in Peru and the changes in findings seen over 12 months while in treatment in Switzerland, 2022 and 2023. Blue arrows highlight the pulmonary consolidation and regression over time. White arrows highlight the enlarged infracarinal lymphnodes and their regression and normalization over time.

We suspected an endemic mycosis infection and ordered serologic testing for *Paracoccidioides* and *Coccidioides*, which were positive with a titer of 1:2. We ordered a complement fixation assay for *Coccidioides* that was positive with a titer of 1:16. The pattern of the *Paracoccidioides* band on the immunodiffusion plate appeared to be a nonidentity reaction, suggesting cross reactivity. To confirm the serologic result, we performed a bronchoalveolar lavage (BAL) of the right lower lobe and endobronchial ultrasound-guided transbronchial needle biopsy of the right upper hilar and subcarinal lymph node. We found an elevated CD4/CD8 ratio in the BAL fluid and a lymphocytic cell count with epithelioid cell granulomas in the biopsy samples. Results of all additional testing for fungi were negative. 

We initiated treatment with itraconazole under the suspected diagnosis of coccidiomycosis or paracoccidioidomycosis. The patient experienced resolution of symptoms, and laboratory parameters returned to reference limits over the following weeks. We conducted a CT scan and serologic examination 6 months after the initiation of treatment. The CT scan revealed almost complete regression of the lung lesion and the lymphadenopathies (Figure 1). The *Coccidioides* serologic testing remained positive, but the *Paracoccidioides* serologic testing was negative. The patient tolerated treatment well, and we made the decision to continue itraconazole for 12 months, after which a final CT scan and serologic testing was performed. The CT scan revealed only a nodular parenchymatous scar remained, and all previous lymphadenopathies had completely disappeared (Figure 1). The *Coccidioides* serologic testing revealed seroreversion and negative results after antifungal therapy concluded. We determined the patient likely contracted coccidioidomycosis on the basis of the clinical manifestations and the serologic results. Coccidioidomycosis is a better fit than paracoccidioidomycosis, and the *Paracoccidioides* serology results are likely because of cross-reactivity between the assays.

*Coccidioides* is a saprotrophic soil fungus and feeds on decayed organic matter. Humans are infected by inhaling airborne arthroconidia when contaminated soil is disturbed (e.g., dust storms, manmade environmental interventions) (*1*). Most cases are reported from the arid and semiarid desert areas of the southwestern United States, but endemic foci also exist in Latin America (*2*). In Peru, coccidioidomycosis is not known to occur, and the single case reported in 1966 is unproven (*3*). Geospatial climate modeling suggests suitable conditions exist along the coast of Peru (*2*), but because there are no previously reported and confirmed coccidioidomycosis cases, we initially limited the serologic testing to histoplasmosis and paracoccidioidomycosis. The patient conducts his field research in the Madre de Dios region, which is an area experiencing heavy mining-related deforestation and desertification (Figure 2). Coccidioidomycosis might surface as an emerging disease in Peru because of manmade environmental changes, as seen in other regions of Latin America (*1*,*4*). In addition, the emergence of coccidioidomycosis in Washington, USA, in 2013 highlights the pathogen’s occurrence in areas previously not considered endemic (*5*–*7*).

**Figure 2:**
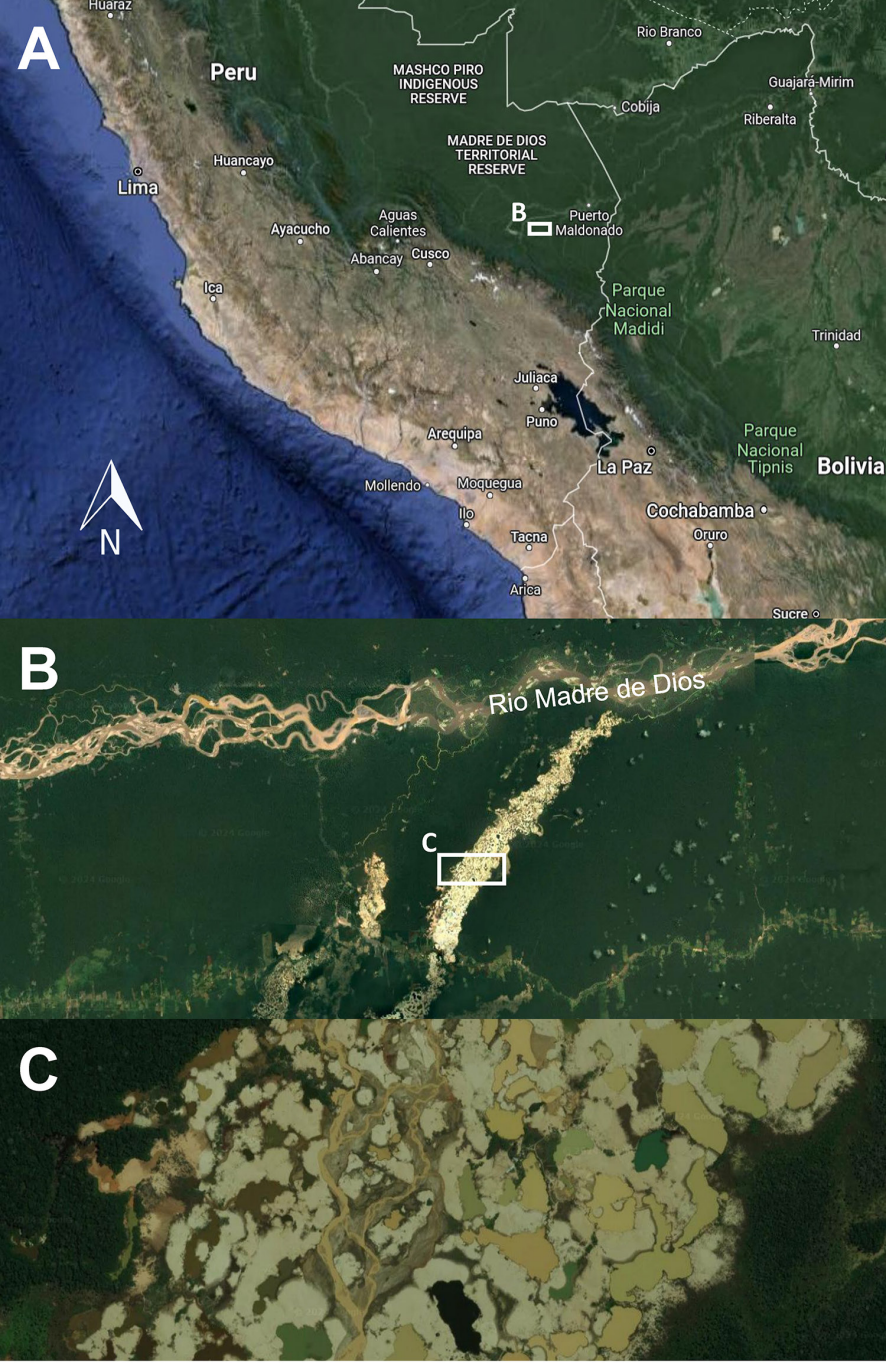
Satellite images of the region in Madre de Dios, Peru, where a patient from Switzerland contracted a suspected case of coccidioidomycosis while conducting field research, 2022. A) Map of southern Peru; B, C) enlargements of the area affected by mining-induced deforestation and desertification, visited by the patient. Images were obtained by using Google Maps (https://maps.google.com).

We believe this case provides evidence that *Coccidioides* might exist in Peru. Coccidioidomycosis could emerge in arid locations in Peru, and clinicians should actively test for it, especially in cases of suspected histoplasmosis or tuberculosis lacking diagnostic confirmation.
